# A reduced vector model predictive controller for a three-level neutral point clamped inverter with common-mode voltage suppression

**DOI:** 10.1038/s41598-024-66013-0

**Published:** 2024-07-02

**Authors:** Ali Bebboukha, Labiod Chouaib, Redha Meneceur, Ahmed Elsanabary, Mohammad Anas Anees, Saad Mekhilef, Ievgen Zaitsev, Mohit Bajaj, Victoriia Bereznychenko

**Affiliations:** 1 Laboratory of Unite Renewable Energy Development Eloued, Department of Mechanical Engineering, University of El Oued, 39000 El Oued, Algeria; 2 Electrical Engineering Department, Faculty of Technology, University of El Oued, 39000 El Oued, Algeria; 3 Department of Electrical Engineering, Port Said University, Port Said, 42526, Egypt; 4 Department of Electrical Engineering, University of Malaya, Kuala Lumpur, Malaysia; 5 School of Science, Computing and Engineering Technologies, Swinburne University of Technology, Melbourne, Australia; 6 Department of Theoretical Electrical Engineering and Diagnostics of Electrical Equipment, Institute of Electrodynamics, National Academy of Sciences of Ukraine, Peremogy, 56, Kyiv-57, 03680 Ukraine; 7 Department of Electrical Engineering, Graphic Era (Deemed to be University), Dehradun, 248002 India; 8 Hourani Center for Applied Scientific Research, Al-Ahliyya Amman University, Amman, Jordan; 9 Graphic Era Hill University, Dehradun, 248002 India; 10 Center for Information-Analytical and Technical Support of Nuclear Power Facilities Monitoring of the National Academy of Sciences of Ukraine, Akademika Palladina Avenue, 34-A, Kyiv, Ukraine

**Keywords:** Grid-connected PV power generation system, Model predictive control, Renewable energy integration, Inverter NPC, Common mode voltage reduction, Voltage vectors, Energy science and technology, Mathematics and computing, Engineering, Electrical and electronic engineering

## Abstract

This paper presents a novel, state-of-the-art predictive control architecture that addresses the computational complexity and limitations of conventional predictive control methodologies while enhancing the performance efficacy of predictive control techniques applied to three-level voltage source converters (NPC inverters). This framework's main goal is to decrease the number of filtered voltage lifespan vectors in each sector, which will increase the overall efficiency of the control system and allow for common mode voltage reduction in three-level voltage source converters. Two particular tactics are described in order to accomplish this. First, a statistical approach is presented for the proactive detection of potential voltage vectors, with an emphasis on selecting and including the vectors that are most frequently used. This method lowers the computational load by limiting the search space needed to find the best voltage vectors. Then, using statistical analysis, a plan is presented to split the sectors into two separate parts, so greatly limiting the number of voltage vectors. The goal of this improved predictive control methodology is to reduce computing demands and mitigate common mode voltage. The suggested strategy's resilience is confirmed in a range of operational scenarios using simulations and empirical evaluation. The findings indicate a pronounced enhancement in computational efficiency and a notable diminution in common mode voltage, thereby underscoring the efficacy of the proposed methodology. This increases their ability to incorporate renewable energy sources into the electrical grid.

## Introduction

The issue of energy scarcity and environmental contamination has emerged as significant and pressing concerns. Consequently, there has been a heightened emphasis on the advancement of sustainable energy sources. Inverters have become essential components of energy conversion systems as a result of the integration of various new energy generating systems and flexible AC transmission devices into the grid^1,2^. The 3L-NPC voltage source inverters offer several advantages over two-level inverters, including lower output harmonics and less semiconductor voltage stress^3^. The emergence of multilevel inverters can be attributed to the introduction of the three-level bidirectional neutral point clamped (NPC) topology, which remains one of the most intriguing configurations now utilized in industrial applications. The NPC employs a solitary direct current (DC) power source, incorporating compact capacitors in the DC-link and a reduced quantity of capacitors in comparison to alternative three-level topologies. Thus far, the NPC has been utilized as a single-phase or three-phase inverter in both standalone and grid-connected operational modes, whereby various techniques have been employed^4^.

In contemporary times, the utilization of model predictive control (MPC) has emerged as a potent mechanism for the control of high-power multilevel converters^5^. Additional notable characteristics of MPC are its ease of design and its ability to withstand changes in system parameters^6^. This control technique is highly advanced and demands substantial computer resources. Nevertheless, it can offer higher performance in specific applications when compared to other control systems^7,8^. (MPC) is a robust technique employed in the control of 3L-NPC. Its main goal is to safely and efficiently operate the system while simultaneously efficiently regulating the inverter's output voltage and frequency to a predefined set point^9^. Using a mathematical model of the inverter system, the MPC algorithm predicts how the output voltage and current will behave in the future. It determines the most effective control inputs that minimize a given cost function within a constrained amount of time using these forecasts^10,11^. In (MPC), the cost function typically includes penalties for deviations from the predefined set point and includes limitations on the inverter's functioning, like voltage and maximum current limits. These factors are taken into account by (MPC), which guarantees that the inverter operates within reasonable bounds and achieves the desired control. By utilizing the most recent measurements and predictions, the algorithm consistently modifies the control inputs, resulting in the establishment of a closed-loop control system^12^. The MPC provides numerous benefits compared to traditional control systems. Moreover, the (MPC) has the ability to effectively manage non-linearities, restrictions, and uncertainties present in the inverter system. The inherent flexibility of this system enables precise and dependable control over the output voltage and frequency. Furthermore, (MPC) makes it easier to include complex control features like reactive power adjustment and harmonic mitigation. These characteristics help to raise the overall effectiveness and performance of the inverter system^13,14^. In recent times, there have been several enhancements implemented on the classical (MPC) approach with the aim of mitigating its complexity and reducing the common-mode voltage (CMV). The CMV holds considerable importance when employing the conventional MPC strategy and its efficacy has been demonstrated. A modified method was proposed in Ref.^15^ Utilizing solely non-zero voltage vectors. Although this strategy has achieved progress, the CMV still experiences the impact of dead time^16^. In addition, it should be noted that the reduction of candidate voltage vectors (VVs) leads to an augmentation in total harmonic distortion (THD). A different approach proposed in the reference^17^ The concept relies on deliberate partial switching, This entails purposefully omitting the transitions between non-adjacent, non-inverting voltage vectors (VVs). It has been shown that this strategy works. That being said, moreover, the implementation of this method is intricate. Authors in reference^18^ proposed a more sophisticated method in contrast to its previous version, which included pre-identifying the sectors to be utilized through a pre-established algorithm. Although this technique successfully decreases the CMV (cost of maintenance) and THD, it is renowned for its intricate procedures and the requirement for substantial mathematical computations for its accurate execution. Modulation techniques or auxiliary circuit schemes might be employed to decrease the CMV^19,20^. Dead time in power electronic inverters causes significant spikes in common mode voltage (CMV) due to all switches being off, resulting in unplanned zero voltage vectors. Mathematically, these spikes can reach up to ±udc/2. By avoiding certain voltage vector switching combinations during dead time, the CMV can be restricted within ±udc/6, reducing spikes, total harmonic distortions (THDs), and current ripples^21^.

The multilayer NPC inverter is plagued by a fundamental problem, which pertains to the balancing of capacitor voltages. Put simply, Stated differently, how to maintain the supposed ripple and a steady (NP) potential^22,23^. A number of problems arise from inadequate control of the NP voltage, such as the inverter output voltage deviating from its reference, the mismatch between the capacitor voltages causing an increase in voltage (THD), and finally, a decline in the inverter's performance^24^. Consequently, in order to control currents and guarantee the voltage balance of the dc link capacitor, this inverter requires a closed-loop control system^25^. Because MPC can effectively handle multi-objective control difficulties, it is commonly recognized as an excellent solution for meeting the control needs of a (3L-NPC)^26^. Its remarkable precision, quick transient response and intrinsic uniqueness make it a desirable substitute control technique for a range of multilevel inverters. This approach is based on the system's discrete-time model, which allows for the prediction of future control variable behavior using the existing switching states. The effectiveness of this approach is contingent upon the utilization of a cost function that incorporates both the current tracking and the error in dc-link capacitor voltages. The inverter is subjected to the selection and application of the ideal switching state that minimizes the cost function at the subsequent sampling instant^27^. Traditional (MPC) necessitates the assessment of the cost function for each switching state of the inverter, resulting in a lengthier execution time. Consequently, this places a significant computational load that consumes a substantial amount of processing resources^28^. The escalation of implementation expenses. Hence, the practical application of (MPC) for multilayer inverter topologies is hindered by the significant computing load and the need for multi-objective control^29^.

In this paper, a refined (MPC) paradigm for a (3L-NPC) inverter is introduced to mitigate computational demands. Initially, a novel algorithm is designed for executing statistical analyses to categorize Non-zero voltage vectors (NZ-VVs) according to their frequency of utilization across different sectors. Subsequent to the analysis, Voltage Vectors (VVs) exhibiting minimal occurrence within each sector are preemptively excluded, with the selection of the eight most prevalent VVs. This innovative approach to MPC adopts a preemptive selection methodology for VVs, aimed at diminishing the computational intensity associated with the evaluation of the cost function. A specific strategy for the preselection of eight Voltage Vectors (8-VVs), grounded in statistical examination, is formulated. This technique capitalizes on all 24 Non-Zero Voltage Vectors (NZ-VVs) available in the 3L-NPC framework to rigorously confine the CMV within the bounds of ±Vdc/6. Moreover, it effectively reduces the computational load imposed by the cost function evaluation and simultaneously lowers (THDs) alongside current fluctuations. To corroborate the efficacy of the articulated strategy, both simulation and empirical findings are discussed. under a spectrum of operational conditions, was meticulously evaluated to validate the proposed method's efficiency, with outcomes delineated accordingly.

## Theoretical foundations and system analysis applications

**Figure 1:**
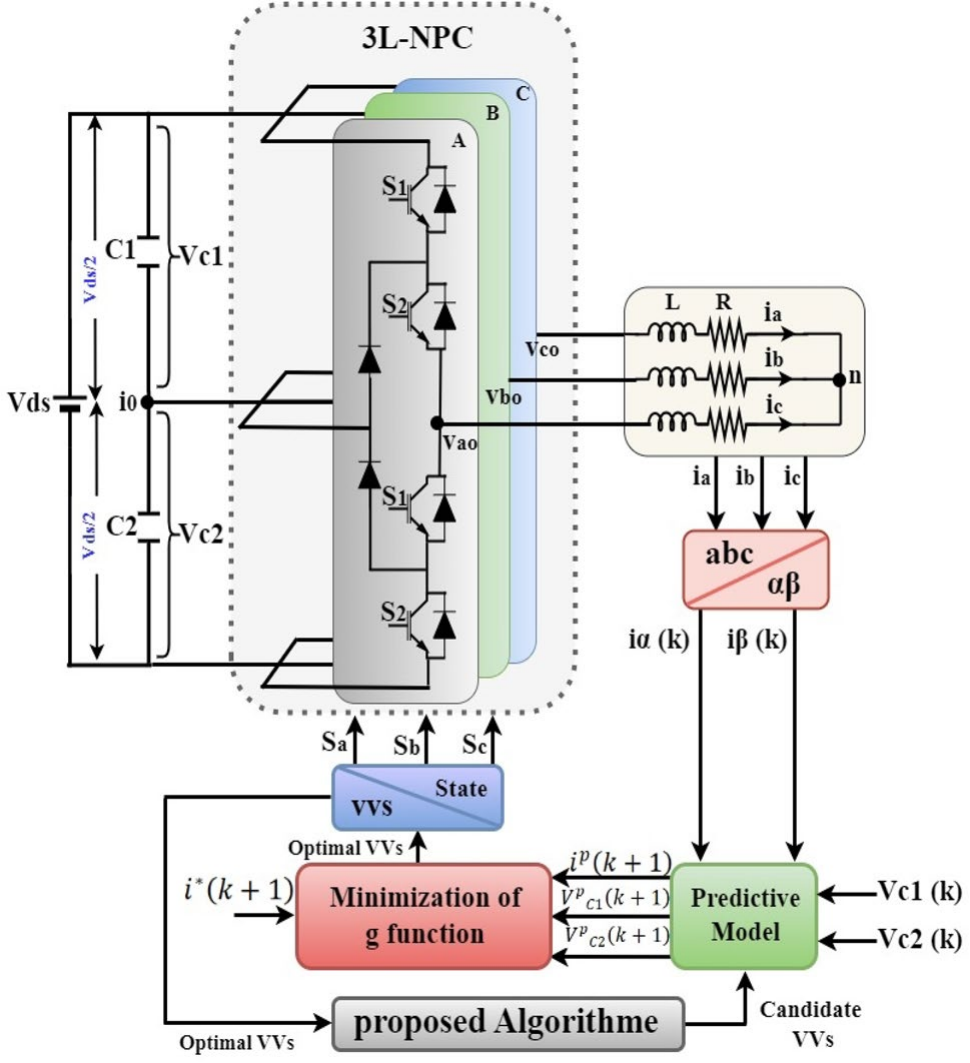
Overall control diagram of the proposed strategy for 3L-NPC connected with load.

The utilization of model predictive control is a prevalent approach in the regulation of voltage source inverters, can make it easier for photovoltaic (PV) systems to integrate their power into the electrical grid. Using a dynamic model of the system, the (MPC) control methodology predicts future behaviour and optimises a control action in a finite amount of time. It is also effective in lowering leakage current that arises from high current-to-voltage^30^. The Fig. 1 a (3L-NPC) inverter, emphasizing its efficiency in converting DC to AC with high power quality. It highlights four semiconductor switches (${\text{S}}_{1}$-${\text{S}}_{4}$) in each leg, controlled to generate the AC output, supported by DC link capacitors (${\text{C}}_{1}$ and ${\text{C}}_{2}$). A predictive model is mentioned for forecasting system states, alongside continuous monitoring of phase currents (${\text{i}}_{\text{a}}$, ${\text{i}}_{\text{b}}$, ${\text{i}}_{\text{c}}$) and voltages (${\text{V}}_{\text{C}1}$, ${\text{V}}_{\text{C}2}$) across the switches^31^. Additionally, the proposed control algorithm optimizes the selection of voltage vectors, thereby reducing computational complexity and mitigating common mode voltage. The figure also depicts the minimization of the cost function, essential for the optimal performance of the inverter, and the effective role of predictive control in enhancing the stability and efficiency of the power conversion process.

## System's mathematical model being investigated

### Examination of the 3L-NPC inverter

**Figure 2:**
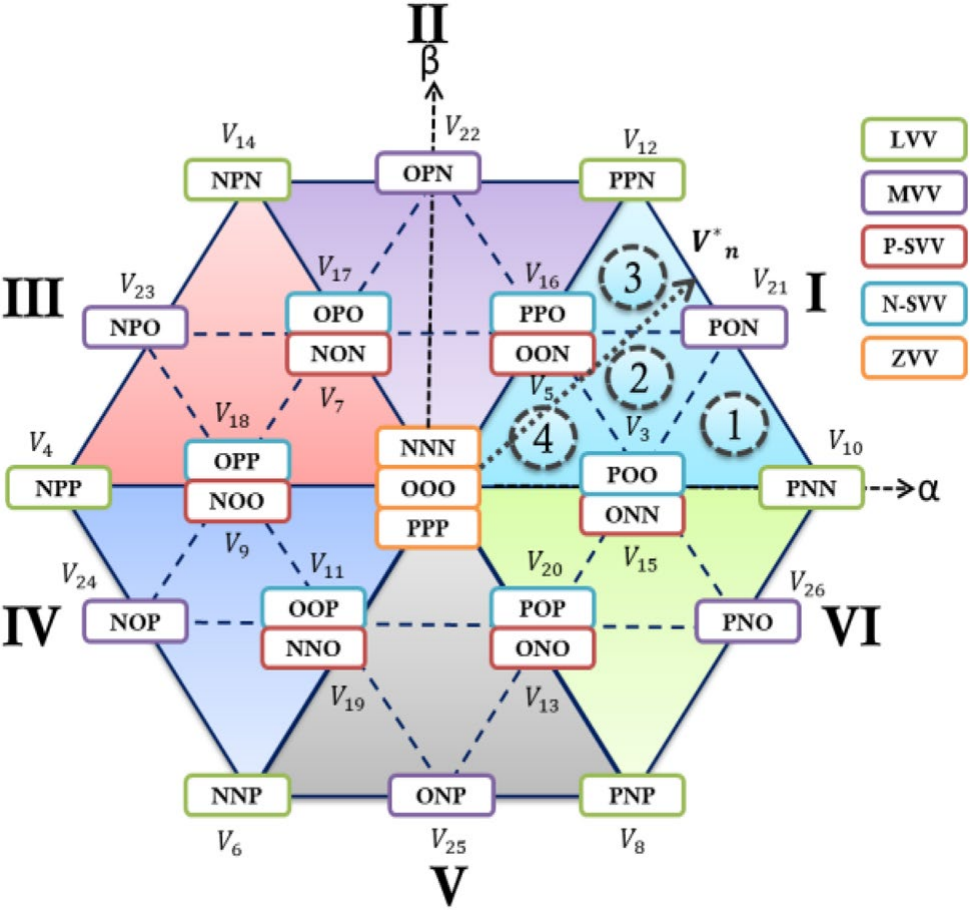
Three-level space vector diagram.

In the power architecture of the Neutral Point Clamped (NPC) inverter, the output voltages, denoted as VP and VN, correspond to the voltages across the upper and lower capacitors of the inverter, respectively. The structure includes three branches, each equipped with four switching devices labeled ${\text{S}}_{\text{X}1}$ through ${\text{S}}_{\text{X}4}$, along with a pair of diodes. The central points of these diodes are linked to the central point between the dc-link capacitors. The operational modes for each phase x of the inverter, where x can be a, b, or c, are detailed in Table 1. In the [P] mode, switches ${\text{S}}_{\text{a}1}$ and ${\text{S}}_{\text{a}2}$ are activated, whereas Sa3 and Sa4 are deactivated, resulting in an output voltage of $\frac{{V}_{dc}}{2}$. Conversely, in the [O] mode, switches ${\text{S}}_{\text{a}2}$ and ${\text{S}}_{\text{a}3}$ are engaged and ${\text{S}}_{\text{a}1}$ and ${\text{S}}_{\text{a}4}$ are disengaged, leading to the clamping of Vdc at zero. In the [N] mode, switches ${\text{S}}_{\text{a}3}$ and ${\text{S}}_{\text{a}4}$ are activated and ${\text{S}}_{\text{a}1}$ and ${\text{S}}_{\text{a}2}$ are deactivated, producing an output voltage of $-\frac{{V}_{dc}}{2}$. These operational modes lead to the generation of 19 unique vectors and eight redundant vectors within the αβ coordinate system. These 27 vectors are categorized into four groups based on their magnitude: zero, small, medium, and large voltage vectors, as illustrated in Fig. 2.

**Table 1:** Switching states of a 3l-npc inverter.

${{\varvec{S}}}_{{\varvec{X}}}$	${{\varvec{S}}}_{{\varvec{X}}1}$	${{\varvec{S}}}_{{\varvec{X}}2}$	${{\varvec{S}}}_{{\varvec{X}}3}$	${{\varvec{S}}}_{{\varvec{X}}4}$	${{\varvec{V}}}_{{\varvec{X}}{\varvec{n}}}$
P	1	1	0	0	${V}_{dc/2}$
O	0	1	1	0	0
N	0	0	1	1	${-V}_{dc/2}$

### Model for predictive analysis of the system in discrete time intervals

The 3 L-NPC inverter models, which include a three-phase resistive-inductive load, can be mathematically represented as:

1 $$\left\{\begin{array}{c}L\frac{{di}_{a}}{dt}={V}_{an}-{Ri}_{a}\\ L\frac{{di}_{b}}{dt}={V}_{bn}-{Ri}_{b}\\ L\frac{{di}_{c}}{dt}={V}_{cn}-{Ri}_{c}\end{array}\right..$$

The application of the Clarke transformation allows for the expression of the 3L-NPC inverter model within the αβ stationary frame:

2 $$\left\{\begin{array}{c}L\frac{{di}_{\alpha }}{dt}={V}_{\alpha n}-{Ri}_{\alpha }\\ L\frac{{di}_{\beta }}{dt}={V}_{\beta n}-{Ri}_{\beta }\end{array}\right..$$

Given the intrinsic correlation between the direct current (DC) link bus voltage and the inverter's output switching configurations, it is feasible to articulate the inverter's terminal voltage as follows:

3 $$\left\{\begin{array}{c}{V}_{an}={V}_{dc}({2S}_{a}-{S}_{b}-{S}_{c})/6\\ {V}_{bn}={V}_{dc}({-S}_{a}+{2S}_{b}-{S}_{c})/6\\ {V}_{cn}={V}_{dc}({-S}_{a}-{S}_{b}+{2S}_{c})/6\end{array}\right. .$$

Equation (3) can be reformulated within the context of a stationary αβ reference framework as follows:

4 $$\left\{\begin{array}{c}{V}_{\alpha n}={V}_{dc}({2S}_{a}-{S}_{b}-{S}_{c})/6\\ {V}_{\beta n}=\sqrt{3}{V}_{dc}({S}_{b}-{S}_{c})/6\end{array}\right..$$

When we talk about the voltage vector, the α and β components are represented by ${V}_{\alpha n}$ and ${V}_{\beta n}$, respectively, while the switching states of the three inverter legs are indicated by the symbols ${S}_{a}$, ${S}_{b}$, and ${S}_{c}$.

#### The forward-Euler method

The forward-Euler method is a straightforward numerical technique used to approximate the derivative of a function in discrete-time modeling. Using the forward-Euler approach.

##### Benefits

Simplicity: The forward-Euler method is easy to implement because it involves basic arithmetic operations. This makes it suitable for quick calculations and understanding fundamental concepts in discrete-time modeling.

Computational Efficiency: It requires minimal computational resources, making it efficient for real-time applications where quick estimations are necessary.

Low Memory Requirements: Since it only requires the current and previous values of the function, it consumes less memory compared to more complex methods.

##### Limitations

Accuracy: The forward-Euler method can be less accurate, especially for systems with rapid changes or non-linearities. The approximation error can accumulate over time, leading to significant deviations from the true solution.

Stability: It can be unstable for stiff equations or when the step size ${T}_{S}$ is not sufficiently small. In such cases, the method might produce oscillatory or diverging solutions.

Error Propagation: The method's error is proportional to the step size ${T}_{S}$. For accurate results, a very small step size is often required, which can increase the computational load and decrease efficiency.

Using the forward-Euler approach, the derivative of the load current can be estimated as follows:

5 $$\frac{di}{dt}\approx \frac{i\left(k+1\right)-i\left(k\right)}{{T}_{s}} .$$

By utilising Eqs. (2) and (5), the projected current of the inverter at the $\left(k+1\right)$ th instant may be expressed.

6 $$\left\{\begin{array}{c}{i}_{\alpha }\left(k+1\right)=\frac{{T}_{s}}{L}({v}_{\alpha n}\left(k\right)-{R}_{\alpha }\left(k\right)){+i}_{\alpha }\left(k\right) \\ {i}_{\beta }\left(k+1\right)=\frac{{T}_{s}}{L}({v}_{\beta n}\left(k\right)-{R}_{\beta }\left(k\right)){+i}_{\beta }\left(k\right)\end{array}\right. .$$

In this context, $k$ represents the sampling time, whereas ${i}_{\alpha }$ and ${i}_{\beta }$ denote the observed currents at the kth sample instant. The determination of the NP current ${i}_{0}$ of the three-level three-phase NPC inverter can be achieved by combining the output currents and switching states, as illustrated in Fig. 1.

7 $${i}_{0}={i}_{C1}-{i}_{C2}=-\left({S}_{a1}+{S}_{a4}\right){i}_{a}-\left({S}_{b1}+{S}_{b4}\right){i}_{b} -\left({S}_{c1}+{S}_{c4}\right){i}_{c} ,$$

The dynamics of capacitor voltages are described through differential equations specific to capacitors.

8 $$\left\{\begin{array}{c}{i}_{C1}=\frac{{i}_{0}}{2}= C\frac{{dV}_{C1}}{dt}\\ {i}_{C2}=\frac{{i}_{0}}{2}= C\frac{{dV}_{C2}}{dt}=C\frac{{d({V}_{dc}-V}_{C2})}{dt}=-C\frac{{dV}_{C1}}{dt}\end{array}\right.$$

Subsequently, the discrete-time representations of the dc-link capacitor voltages are derived through the implementation of the forward Euler method, as outlined below:

9 $$\left\{\begin{array}{c}{V}_{C1}\left(k+1\right)= {V}_{C1}\left(k\right)+\frac{1}{C}{i}_{C1}\left(k\right){T}_{s}={V}_{C1}\left(k\right)+\frac{1}{C}{i}_{0}\left(k\right){T}_{s}\\ {V}_{C2}\left(k+1\right)= {V}_{C2}\left(k\right)+\frac{1}{C}{i}_{C2}\left(k\right){T}_{s}={V}_{C2}\left(k\right)+\frac{1}{C}{i}_{0}\left(k\right){T}_{s}\end{array}\right..$$

In the context of the 3L-NPC inverter, the traditional (MPC) approach is designed with dual goals: firstly, to facilitate swift and precise tracking of current, and secondly, to ensure the balancing of the inverter's neutral point (NP) voltage through the employment of weighting factors. Consequently, the cost function for the conventional MPC, which is predicated upon a predictive model of current, is articulated as follows:

10 $$g=\left|{i}_{\alpha }^{*}\left(k+1\right)-{i}_{\alpha }\left(k+1\right)\right|+\left|{i}_{\beta }^{*}\left(k+1\right)-{i}_{\beta }\left(k+1\right)\right|+{\lambda }_{dc}\left|{V}_{C1}\left(k+1\right)-{V}_{C2}\left(k+1\right)\right| .$$

The selection of weighting factors in the cost function for a three-level NPC inverter involves determining the relative importance of current tracking versus NP voltage balancing. This is primarily guided by system priorities: a lower weighting factor (${\lambda }_{dc}$) prioritizes fast and accurate current tracking, while a higher ${\lambda }_{dc}$ emphasizes NP voltage balancing. Initially, an empirical value for ${\lambda }_{dc}$​ is chosen and iteratively adjusted based on performance. Simulations and optimization algorithms can model the inverter's behavior under various conditions, helping to refine the weighting factor.

The current references in the αβ reference frame, denoted as ${i}_{\alpha \beta }^{*}\left(k+1\right)$, can be derived using the second-degree Lagrange extrapolation method, as as follows.

11 $$\left\{\begin{array}{c}{i}_{\alpha }^{*}\left(k+1\right)=3{i}_{\alpha }^{*}\left(k\right)-{3i}_{\alpha }^{*}\left(k-1\right)+{i}_{\alpha }^{*}\left(k-2\right) \\ {i}_{\beta }^{*}\left(k+1\right)=3{i}_{\beta }^{*}\left(k\right)-{3i}_{\beta }^{*}\left(k-1\right)+{i}_{\beta }^{*}\left(k-2\right)\end{array}\right..$$

Drawing upon Eqs. (6), (9), and (10), it becomes evident that the determination of an optimal switching state for the 3L-NPC inverter via traditional (MPC) necessitates twenty-seven predictions each for current and Neutral Point (NP) voltage, alongside twenty-seven evaluations of the cost function. Consequently, this entails a requisite of eighty-one iterations per sampling period, thereby augmenting the computational load. Additionally, the nuanced and labor-intensive process of heuristically selecting a weighting factor to ensure the preservation of proportional significance amongst multiple control objectives, specifically between current tracking and NP voltage regulation further complicates the operational framework.

## Proposed strategy

The proposed strategy's overall control diagram and the 3L-NPC switching states connected to the load are shown in Fig. 1. In each sector, the modified MPC method aims to only reduce the candidate compounds number to eight; it is divided according to the reference beam length into two parts, based on statistical calculations. This method is called the 8-VVs method for simplicity.

The methodology of the 8-VVs approach is executed through a series of systematic phases. Initially, Z-VVs are eliminated from the process. Subsequently, NZ-VVs are deployed throughout the entire cycle to guarantee extensive encompassment across all sectors. In the following stage, the dominant VVs within each sector are systematically classified to identify those with frequent utilization. Thereafter, consideration is given solely to the top eight VVs in each segment, as identified by the aforementioned classification process. These optimal VVs are then applied at every phase. Moreover, this strategy exclusively utilizes the eight identified VVs, continually excluding previously overlooked VVs, provided that the margin of error remains minimal, thereby ensuring precision in outcomes. This procedure is replicated with each iteration, with ${\boldsymbol{\Delta }}_{{\varvec{i}}}$ representing the percentage modification in the length of reference vectors. In instances of significant error, VVs that were previously eliminated are reintroduced, and all VVs are reconsidered as potential candidates, thereby circumventing any potential failure of the method.

The algorithm for voltage vector classification uses the frequency of each vector's utilization in different sectors. Initially, (NZ-VVs) are applied using (MPC). A frequency analysis identifies the top eight vectors per sector, reducing computational intensity by focusing on these prevalent vectors. The algorithm ensures optimal performance by evaluating and adjusting (THD) and (RMSec). If these exceed thresholds, further adjustments are made. The method assumes frequently used vectors represent optimal control actions, ensuring efficient and accurate inverter operation.

A graphical representation of this process is depicted in Fig. 1.

## Statistics results

**Figure 3:**
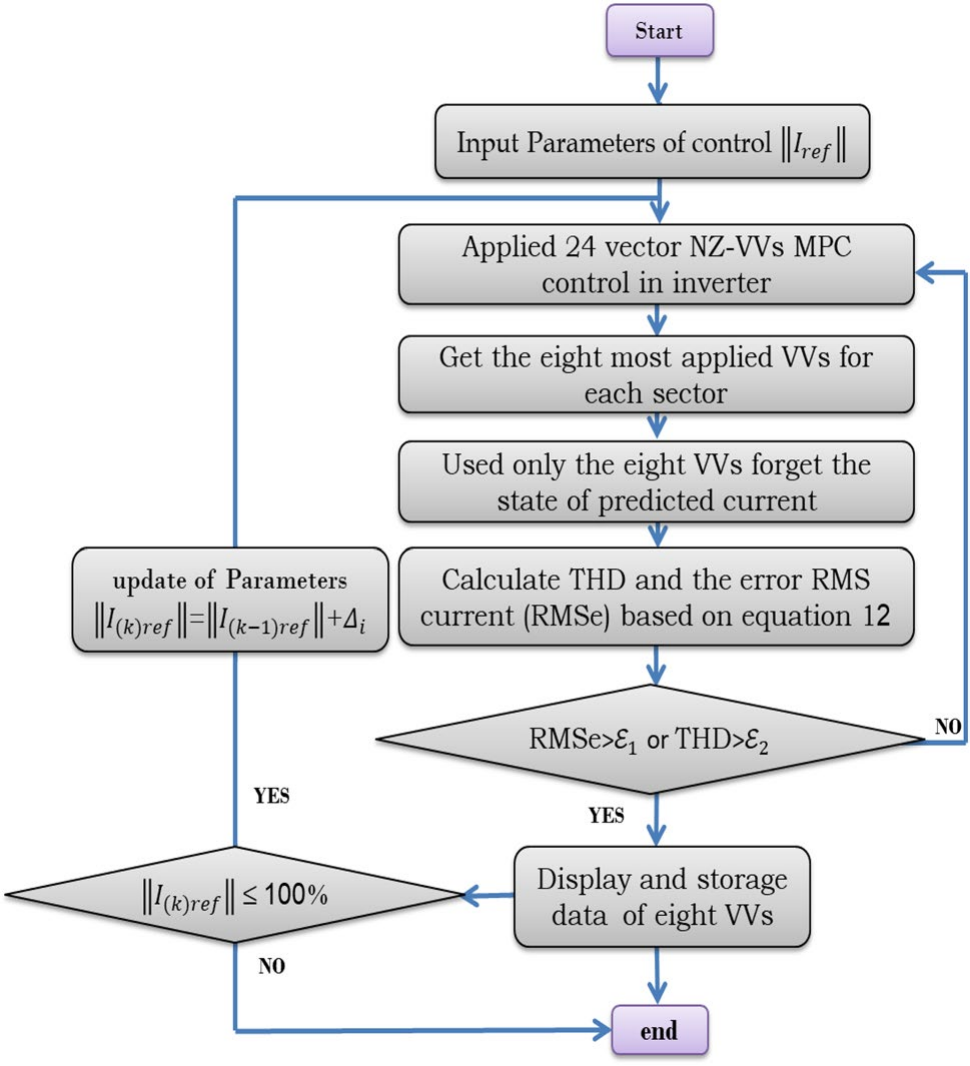
Execution processes of the proposed method.

**Figure 4:**
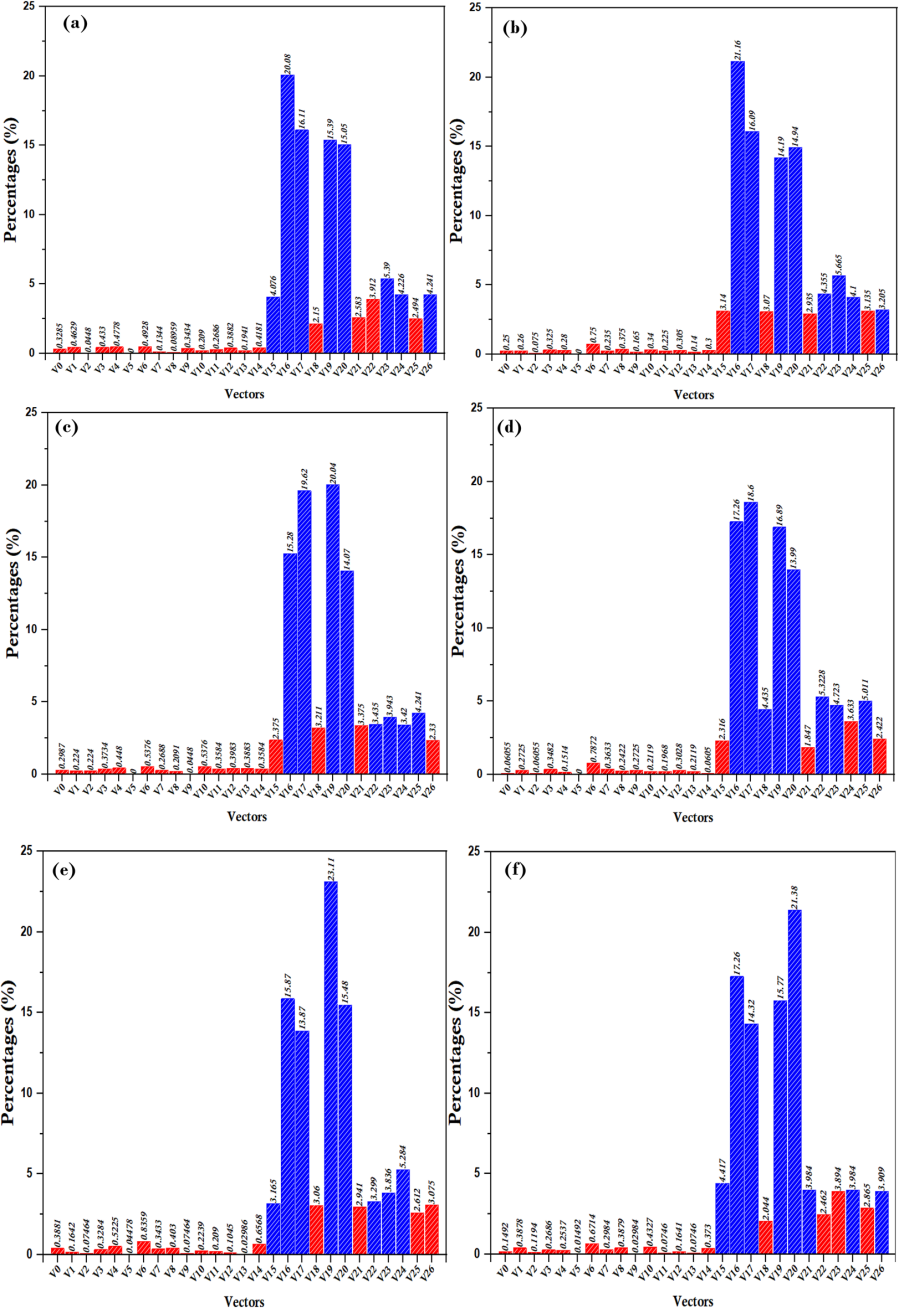

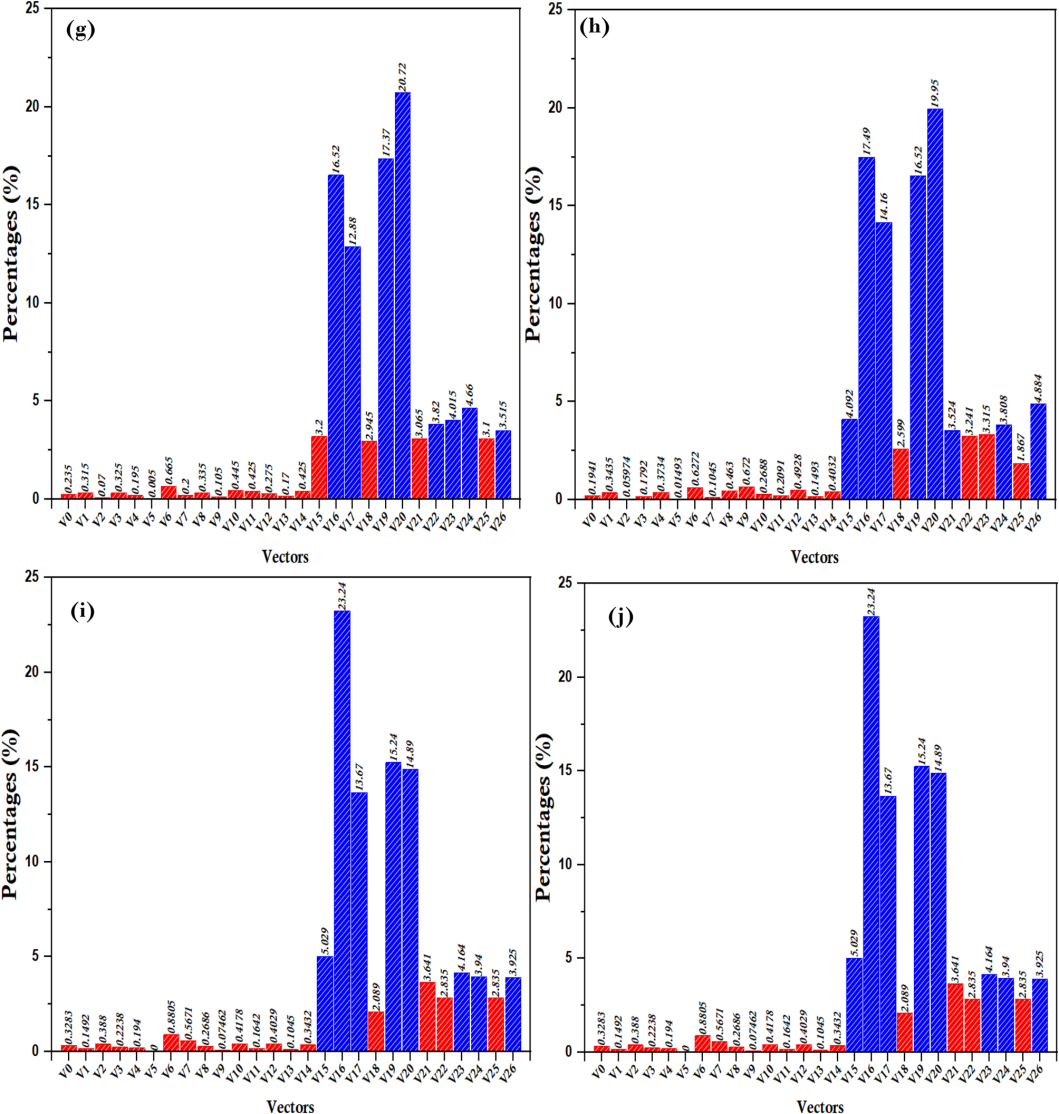
The selection ratio of variable vectors (VVs) at each change ${\boldsymbol{\Delta }}_{{\varvec{i}}}$ relative magnitude of reference $\Vert {{\varvec{I}}}_{({\varvec{k}}){\varvec{r}}{\varvec{e}}{\varvec{f}}}\Vert$**.**

In the most widely used vector selection statistics, a systematic evaluation methodology was used to ascertain the optimal beam based on the length of the reference vector $\Vert {{\varvec{I}}}_{{\varvec{r}}{\varvec{e}}{\varvec{f}}}\Vert$ by implementing the proposed method shown in Fig. 3. Using the variable ${\boldsymbol{\Delta }}_{{\varvec{i}}}$​to set the reference length ratio $\Vert {{\varvec{I}}}_{({\varvec{k}}){\varvec{r}}{\varvec{e}}{\varvec{f}}}\Vert$, the evaluation proceeded iteratively, with each iteration setting ${\boldsymbol{\Delta }}_{{\varvec{i}}}$ by 10%, thus capturing the results associated with each change. This iterative approach facilitated a comprehensive exploration of the relationship between the length of the reference vector $\Vert {{\varvec{I}}}_{({\varvec{k}}){\varvec{r}}{\varvec{e}}{\varvec{f}}}\Vert$ and the selection of the most commonly used vectors. The process yielded insights shown in Fig. 4, providing a visual representation of the results obtained through iterative statistics.

**Figure 5:**
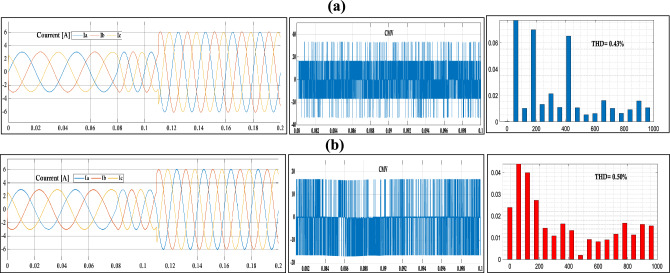
Simulations pertaining to the output current along with the common-mode voltage (CMV) and total harmonic distortion (THD). (**a**) (NZ-VVs), and (**b**) (8-VVs).

The most commonly used vectors are selected for example as follows:

- In (${\boldsymbol{\Delta }}_{{\varvec{i}}}$=10%), the VVs V15, V16, V17, V19, V20, V23, V24, and V26 are the most applied, as shown in Fig. 5a.
- In (${\boldsymbol{\Delta }}_{{\varvec{i}}}$=20%), the VVs V16, V17, V19, V20, V22, V23, V24, and V26 are the most applied, as shown in Fig. 5b.
- In (${\boldsymbol{\Delta }}_{{\varvec{i}}}$=30%), the VVs V16, V17, V19, V20, V22, V23, V24, and V25 are the most applied, as shown in Fig. 5c.

## Simulation results

In this section, the efficacy of the newly introduced strategy employing 8-VVs is assessed through comparative simulations with the traditional NZ-VVs approach. These simulations were executed using the MATLAB/Simulink environment to ascertain the impact of the advanced Model Predictive Control MPC technique on diminishing the common mode voltage CMV. The simulation duration was established at 0.2 seconds, with the magnitude of the output reference current adjusted from 2 Amperes to 1 Ampere, whilst maintaining a constant reference frequency of 50 Hz. Figure 5 illustrates the outcomes, showcasing the CMV levels attained through the implementation of various strategies, including the conventional NZ-VVs method and the 8-VVs strategy proposed herein.

The simulation results indicate that the proposed method (8-VVs) significantly outperforms the alternative method (NZ-VVs) in terms of current tracking, common-mode voltage (CMV) stability, and total harmonic distortion (THD). The proposed method achieves smooth and sinusoidal output currents with a THD of 0.50%, which, while slightly higher than the 0.43% THD of the alternative method, still demonstrates effective waveform quality. Additionally, the proposed method maintains a more stable CMV with fewer significant fluctuations, enhancing the reliability and lifespan of the inverter by reducing electromagnetic interference and component stress. These improvements imply that the proposed method offers more efficient and reliable inverter performance, making it a superior choice for practical applications requiring high-quality power conversion and stable operation. Changes in frequency from 25 to 50 Hz, as depicted in the simulations (Fig. 5), further highlight the robustness of the proposed method. The method consistently maintains superior performance across this frequency range, ensuring reliable and efficient operation under varying conditions. This frequency adaptability is crucial for applications where operating conditions are not constant, further solidifying the proposed method's practicality and effectiveness in real-world scenarios.

**Figure 6:**
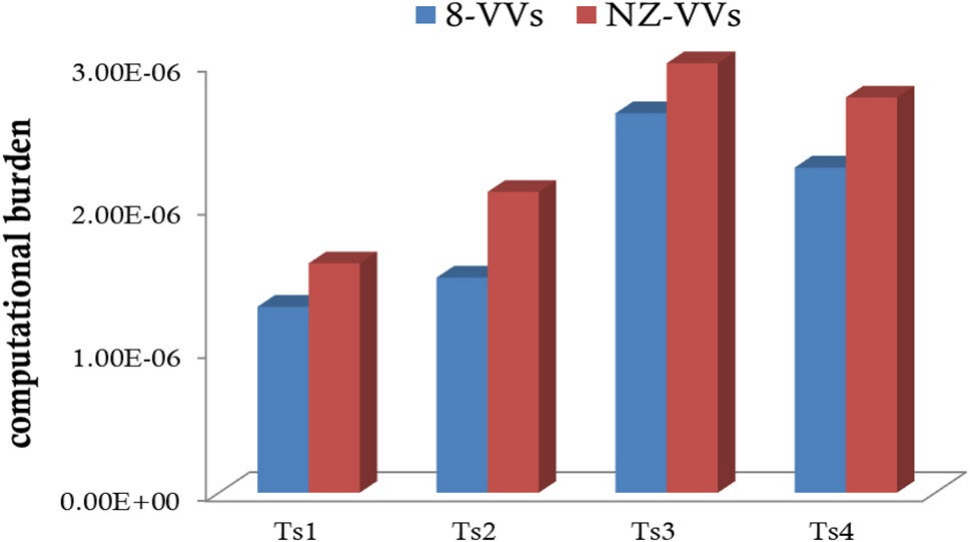
Comparison of computational burden for 8-VVs and NZ-VVs methods across different sampling times (Ts) in predictive algorithms.

The provided Fig. 6 compares the computational burden of two methods, 8-VVs and NZ-VVs, across four different sampling times (Ts1, Ts2, Ts3, Ts4) for the predictive algorithm. Quantitative analysis reveals that the 8-VVs method consistently exhibits a lower computational burden compared to the NZ-VVs method. For instance, during Ts2, the computational burden for 8-VVs is approximately 1.50e–6 seconds, whereas for NZ-VVs, it reaches around 2.00e–6 seconds, indicating a significant reduction of 0.50e–6 seconds. Similar reductions are observed in other sampling times, such as Ts3 and Ts4, where 8-VVs consistently outperform NZ-VVs. These results demonstrate that the 8-VVs method offers a substantial reduction in computational burden, thereby enhancing computational efficiency and making it a more optimal choice for applications requiring lower computational resources in predictive algorithms.

## Experimental evaluations

**Figure 7:**
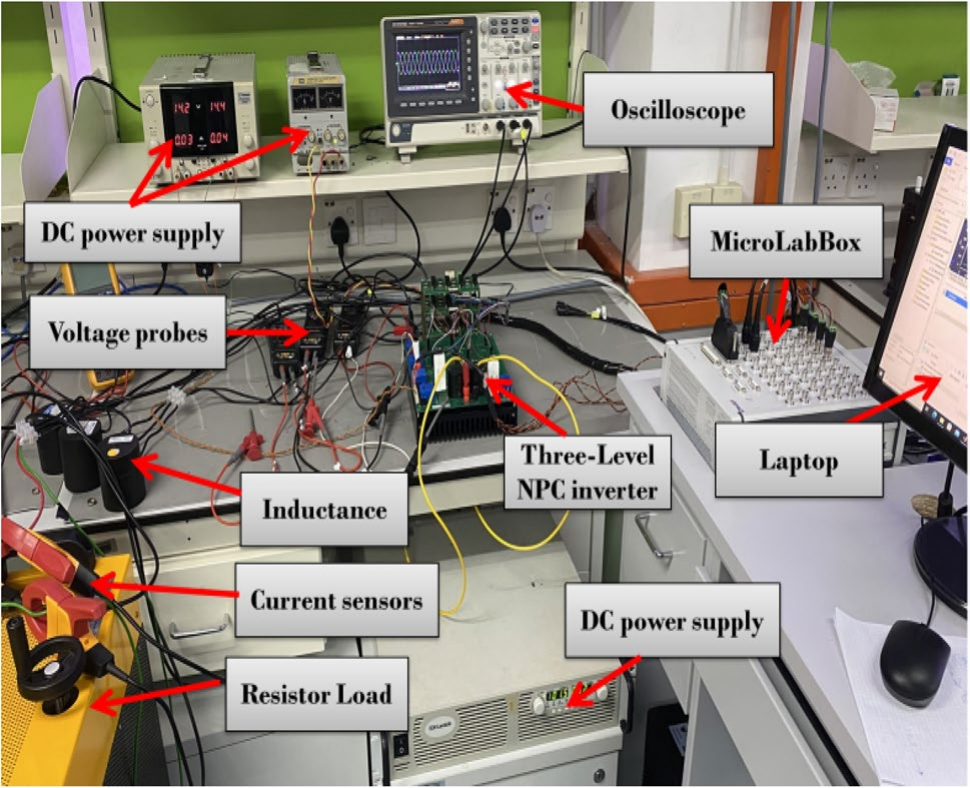
Experimental setup used for performance evaluation.

The study employs an experimental setup incorporating a three-level Neutral Point Clamped (NPC) inverter, which derives its power from a direct current (DC) source and delivers it to a resistive-inductive (R-L) load. The construction of the inverter involves the utilization of twelve FGH40N60 MOSFETs and six SiC C4D30120D diodes, provided by CREE Inc. For the purpose of implementing various Model Predictive Control (MPC) strategies, a MicroLabBox board is deployed. This board facilitates the generation of switching signals for the semiconductor devices, achieved through twelve digital outputs from the MicroLabBox system. To precisely observe the output phase currents of the inverter and the DC-link voltages, the methodology incorporates the utilization of three LEM LA-25P transducers for current measurement and two LEM LV-25NP transducers for voltage measurement. The signal acquisition from these sensors is executed through five analog-to-digital converter (ADC) inputs located on the MicroLabBox, ensuring precise data collection for analysis. To ascertain the efficacy of the two voltage-based 8-VVs and NZ-VVs predictive control mechanisms, two experimental evaluations are conducted. These evaluations aim to rigorously assess the steady-state and dynamic capabilities of all contending model predictive control (MPC) algorithms. Figure 7 illustrates the configuration of the experiment, whereas Table 2 enumerates the primary parameters utilized in the experiment.

**Table 2:** Experimental parameters.

Parameter	Numerical value
DC-link voltage [V]	100
Reference frequency [Hz]	50
Inductance load [mH]	12
Resistance load [Ω]	10
Capacitor (C1, C2) [µF]	3300

### Experimental data analysis

**Figure 8:**
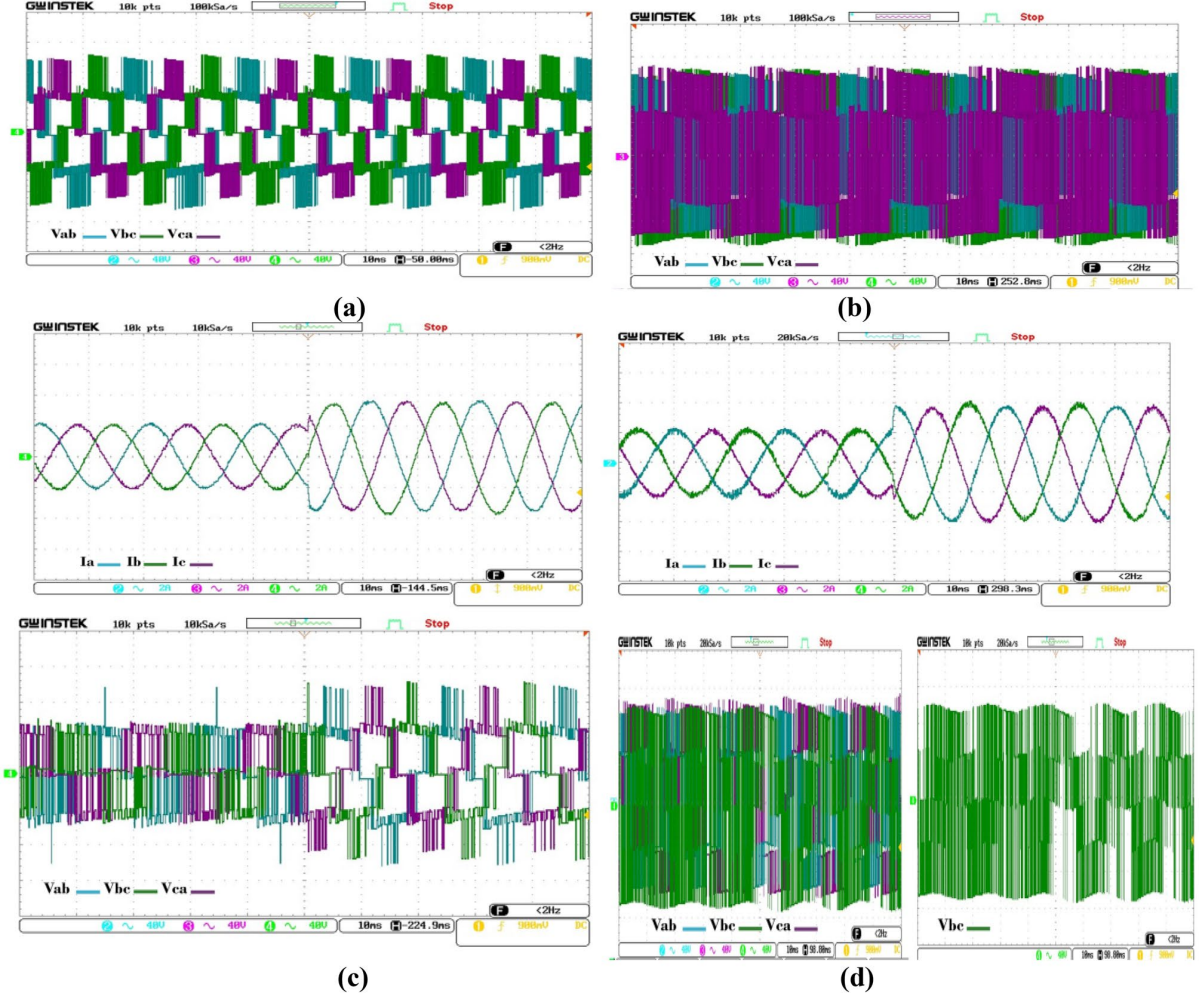
Empirical findings about the performance at a stable state (**a**) The traditional approach (NZ-VVs); (**b**) the technique involving 8-VVs; and Experimental findings of the dynamic performance when the current is increased from 2 to 4 A. (**c**) The standard strategy, known as NZ-VVs; (**d**) The strategy of 8-VVs.

To meticulously examine the transient behaviors exhibited by two distinct control methodologies, namely (NZ-VVs) and (8-VVs), an exhaustive and methodical analytical study was conducted. This study comprehensively evaluated both conventional and cutting-edge algorithms under a wide array of operational scenarios and testing milieus. A crucial aspect of our analysis was the detailed investigation of the output current waveforms (Ia, Ib, Ic) generated by the (3L-NPC) inverter, a key feature depicted in Fig. 8. The data obtained from these empirical evaluations indicate that the predictive control approach, as implemented by the 8-VVs methodology, exhibits superior dynamic performance, demonstrating remarkable proficiency in managing both typical and intricate dynamic conditions effectively.

In an effort to provide a deeper insight into the relative dynamic efficiencies of these control strategies, simulation experiments were elaborately designed and executed with precision. The aim of these simulations was to scrutinize the dynamic performance characteristics of an array of both traditional and advanced predictive control mechanisms, particularly in response to a sudden change in reference current from 2 to 4 A a scenario illustratively captured in Fig. 8. Furthermore, our investigations unveiled that predictive controllers based on database architecture not only respond swiftly to dynamic alterations but also safeguard the structural integrity of the database. This feature is of utmost significance, highlighting the controllers' capability to sustain optimal performance levels without detriment to the essential database structure. Figure 8 (c), (d) presents a comparative analysis of the dynamic voltage responses of the NZ-VVs and 8-VVs (Vab, Vbc, Vca) consequent to the reference current increment from 2 to 4 A. While the traditional NZ-VVs scheme may offer enhanced steady-state performance over the proposed control scheme, the dynamic response facilitated by the novel NZ-VVs-8 configuration is significantly swifter than that offered by the conventional NZ-VVs setup.

**Figure 9:**
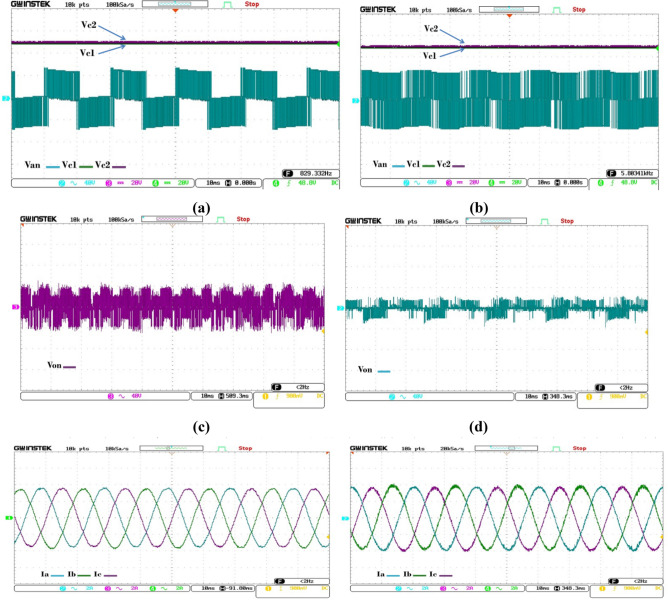
Experimental results of capacitor voltages (Vc1, Vc2) (**a**) The traditional approach (NZ-VVs); (**b**) the technique involving 8-VVs; and Experimental findings of the dynamic performance when the current is increased from 2 to 4 A. (**c**) The standard strategy, known as NZ-VVs; (**d**) The strategy of 8-VVs.

**Figure 10:**
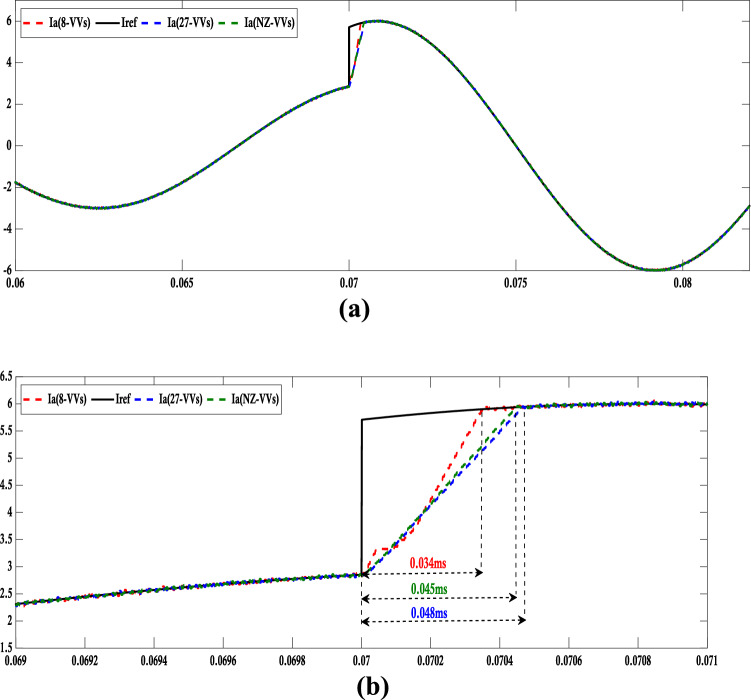
(**a**) Evaluation of dynamic-state performance via simulation for conventional MPC: Comparing (27-VVs), (NZ-VVs), and (8-VVs). (**b**) Detailed examination of the step-change segment.

An examination of Fig. 9 elucidates the occurrence of a high-amplitude Common Mode Voltage (CMV) when the inverter operates under the control mechanism of (NZ-VVs). The application of eight-voltage vectors (8-VVs) leads to a significant suppression of the CMV, indicating a more favorable operational condition for reducing common mode disturbances. Further scrutiny is warranted by Fig. 10, which delineates the voltage profiles corresponding to Vc1, Vc2, and Van. It is noteworthy that the voltage of Van, alongside the neutral point (NP) voltage-balancing parameters (Vc1 and Vc2), are preserved with remarkable consistency, nearly approximating 50 V. This is achieved with a minimal deviation, not exceeding a 2 V, attributable primarily to the presence of voltage ripple. This subtle variance underscores the efficacy of the employed voltage vector strategy in maintaining voltage stability and minimizing fluctuations, thereby enhancing the overall performance and reliability of the inverter system.

The controller, characterized by an 8-variable vector (8-VVs), exhibits the most rapid dynamic response and achieves the minimum settling time of 0.034 milliseconds when contrasted with alternative algorithms.

### Performance tests

To ascertain the effectiveness of the proposed methodology, the operational capabilities of the inverter are evaluated under a variety of conditions. This evaluation entails the abrupt alteration of parameters. The metric utilised for evaluating performance is the Total Harmonic Distortion (THD) of the output current.

The selection of The current error as measured by the root mean square (RMS) as the metric of choice is predicated on its aptitude for accurately assessing the instantaneous conformity of the actual current to its reference counterpart. This evaluation metric can be determined through the following calculation:

12 $$\text{RMSe}=\sqrt{\frac{{\int }_{{\text{T}}_{\text{i}}}^{{\text{T}}_{\text{f}}}{\left({\text{i}}^{*}\left(\text{t}\right)-\text{i}\left(\text{t}\right)\right)}^{2}\text{dt}}{{\text{T}}_{\text{f}}-{\text{T}}_{\text{i}}}}$$

The reference current is denoted as ${\text{i}}^{*}\left(\text{t}\right)$, while the measured current is represented as $\text{i}\left(\text{t}\right)$. The variables ${\text{T}}_{\text{f}}$ and ${\text{T}}_{\text{i}}$ represent the upper and lower limits, respectively, for the selected time interval.

**Figure 11:**
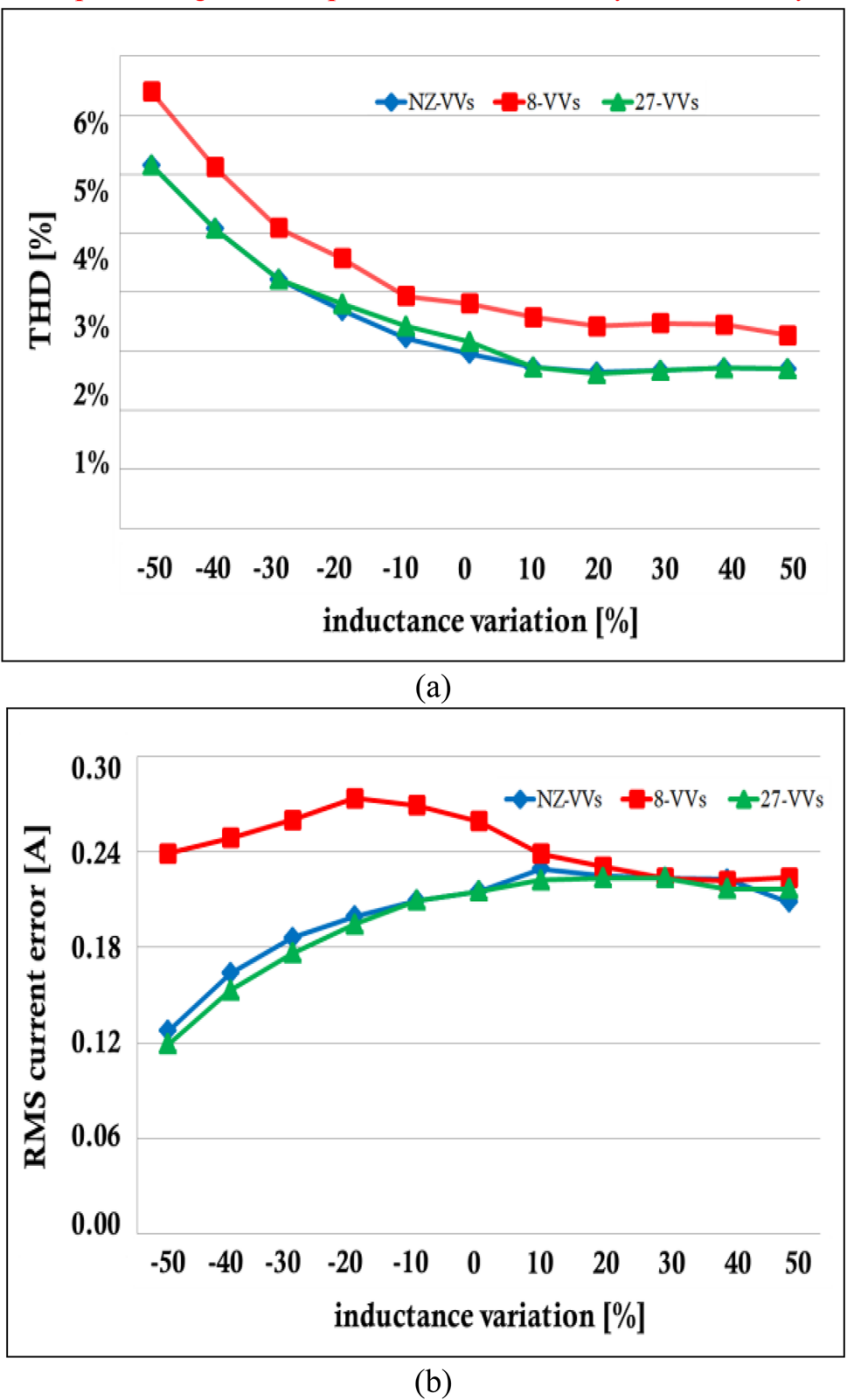
Sensitivity of the conventional MPC (27-VVs) and (NZ-VVs),(8-VVs) predictive controllers with varying inductor arm configurations. The THD percentage.The root mean square (RMS) current error.

An examination of the impact of inductance variations on the performance of an inverter was conducted through an analysis of total harmonic distortion (THD) and the root mean square (RMS) current error in phase-a of the output current. The study specifically focused on the response of the inverter's phase-a output current to inductance alterations, which ranged from a decrease of 50% to an increase of 50%. According to the findings depicted in Fig. 11a, with a 50% decrease in inductance, the THD for 8-VVs is around 5%, while for 27-VVs it is around 4%. As inductance increases to 50%, the THD for both strategies decreases, with 8-VVs reaching approximately 2.5% and 27-VVs around 2%. Despite the reduction in the selection pool, the 8-VVs strategy maintains a comparable level of performance to the 27-VVs strategy, ensuring reliability and stability by leveraging the inductance's low-pass filter effect and the convergence in THD performance at higher inductance values, and it is evident that inductance fluctuations significantly influence THD levels. The analysis revealed that an escalation in inductance correlates with a reduction in THD across all examined strategies, attributed to the inductance's role as a low-pass filter. In terms of the RMS current error's sensitivity to changes in inductance, the 8-VVs strategy demonstrated commendable efficacy, as illustrated in Fig. 11b, albeit being slightly surpassed by the traditional 27-VVs strategy. It is important to highlight that the performance disparity among the strategies narrows with an increase in inductance value, indicating a convergence in efficiency across different methodologies.

The trade-offs between reduced computational load and control precision involve selecting the top eight most frequently used voltage vectors to enhance computational efficiency and speed up MPC cost function evaluation. This reduction in computational intensity ensures faster processing while maintaining control precision by confining CMV within ±Vdc/6, lowering THD, and minimizing current fluctuations. Thus, computational demands are reduced without compromising inverter performance reliability and accuracy.

## Conclusions

This research paper introduces an enhanced model predictive control (MPC) architecture for 3L-NPC inverters, aiming to significantly reduce the computational demands associated with traditional MPC approaches, strategically refining the selection of candidate (VVs) and eliminating (Z-VVs), the paper proposes a narrowed selection of eight (NZ-VVs) from the traditional twenty-four In the most widely used vector selection statistics. This bespoke algorithm, through rigorous statistical analyses, categorizes the VVs used in each interval, laying a robust theoretical groundwork for the architecture of the proposed approach. These advancements are validated through experimental evaluations and simulations, demonstrating that the novel strategy not only mitigates significantly reduce the computational but also maintains a tolerable (THD) level, thereby enhancing the efficiency of the 3L-NPC inverter. The results demonstrate substantial improvements in computational efficiency and a significant reduction in common mode voltage, thereby affirming the robustness and practical viability of the proposed method in enhancing the inverter's performance and reliability.

The methodology for (3L-NPC) inverters presents certain limitations, particularly in terms of adaptability to dynamic conditions. Future research should prioritize the development of real-time adaptive algorithms, investigation of long-term stability, and extensive testing to improve performance and reliability. Moreover, this study contributes to addressing some inherent faults in the inverter.

## Data Availability

The datasets used and/or analysed during the current study available from the corresponding author on reasonable request.
